# *N*-Hy­droxy­pyridine-4-carboxamide

**DOI:** 10.1107/S1600536811025566

**Published:** 2011-07-06

**Authors:** Qingkun Wu, Handong Yin

**Affiliations:** a College of Chemistry and Chemical Engineering, Liaocheng University, Shandong 252059, People’s Republic of China

## Abstract

The title compound, C_6_H_6_N_2_O_2_, is approximately planar with an r.m.s. deviation for the non-H atoms of 0.052 Å. In the crystal, a two-dimensional array in the *bc* plane is stabilized by O—H⋯N and N—H⋯O hydrogen bonds.

## Related literature

For background to the coordination chemistry of hydroxamic acid derivatives, see: Codd (2008 ▶). For related structures, see: Wang *et al.* (1988 ▶); Makhmudova *et al.* (2000 ▶); Golenya *et al.* (2007 ▶). 
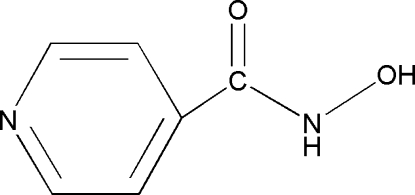


## Experimental

### 

#### Crystal data

C_6_H_6_N_2_O_2_
*M* *_r_* = 138.13
Monoclinic, 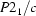

*a* = 4.8765 (5) Å
*b* = 13.4476 (16) Å
*c* = 9.6656 (11) Å
β = 99.579 (1)°
*V* = 625.01 (12) Å^3^
*Z* = 4
Mo *K*α radiation
μ = 0.11 mm^−1^
*T* = 298 K
0.35 × 0.24 × 0.15 mm

#### Data collection

Bruker SMART CCD area-detector diffractometer
Absorption correction: multi-scan (*SADABS*; Sheldrick, 1996 ▶) *T*_min_ = 0.961, *T*_max_ = 0.983
3030 measured reflections
1092 independent reflections
769 reflections with *I* > 2σ(*I*)
*R*_int_ = 0.024

#### Refinement

*R*[*F* ^2^ > 2σ(*F* ^2^)] = 0.039
*wR*(*F* ^2^) = 0.115
*S* = 1.05
1092 reflections
92 parameters
H-atom parameters constrained
Δρ_max_ = 0.15 e Å^−3^
Δρ_min_ = −0.16 e Å^−3^

### 

Data collection: *SMART* (Siemens, 1996 ▶); cell refinement: *SAINT* (Siemens, 1996 ▶); data reduction: *SAINT*; program(s) used to solve structure: *SHELXS97* (Sheldrick, 2008 ▶); program(s) used to refine structure: *SHELXL97* (Sheldrick, 2008 ▶); molecular graphics: *SHELXTL* (Sheldrick, 2008 ▶); software used to prepare material for publication: *SHELXTL*.

## Supplementary Material

Crystal structure: contains datablock(s) I, global. DOI: 10.1107/S1600536811025566/tk2754sup1.cif

Structure factors: contains datablock(s) I. DOI: 10.1107/S1600536811025566/tk2754Isup2.hkl

Supplementary material file. DOI: 10.1107/S1600536811025566/tk2754Isup3.cml

Additional supplementary materials:  crystallographic information; 3D view; checkCIF report

## Figures and Tables

**Table 1:** Hydrogen-bond geometry (Å, °)

*D*—H⋯*A*	*D*—H	H⋯*A*	*D*⋯*A*	*D*—H⋯*A*
O1—H1⋯N2^i^	0.82	1.92	2.721 (2)	166
N1—H2⋯O2^ii^	0.86	2.01	2.844 (2)	162

Symmetry codes: (i) 
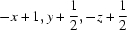
; (ii) 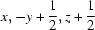.

**Fig. 1.**
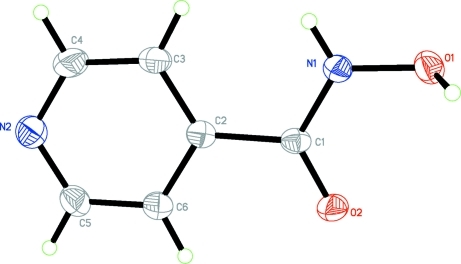
The molecular structure of (I), showing atom-labelling and 50% probability displacement ellipsoids.

**Fig. 2.**
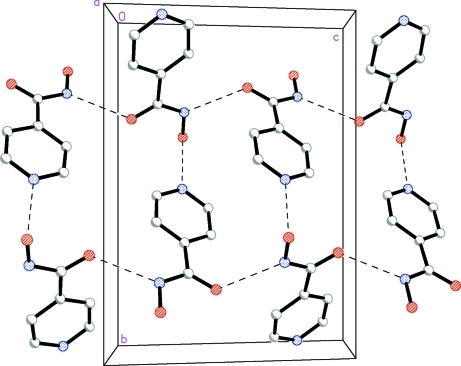
The supramolecular 2-D array in the bc plane stabilised by N—H···O and O—H···N hydrogen bonds (dashed lines).

## supplementary crystallographic information

### Comment

Hydroxamic acid, R1C(═O)N(R2)OH (R1 = alkyl/aryl; R2 = alkyl/aryl or H), relevant to chemical biology, coordinate a wide variety of metal ions predominantly as the monoanionic hydroxamato or as a dianionic (R2 = H) hydroximato *O*,*O*-bidentate chelate (Codd, 2008). To the best of our knowledge, while a large number of transition metal derivatives with hydroxamic acids have been reported, organoantimony complexes with hydroxamic acids are limited. To further extend this field and to construct novel structures of organoantimony, we choose to investigate reactions involving the title compound (I). Herein, we present its crystal structure determination.

In (I), the non-hydrogen atoms are in the same plane (Fig. 1) with the rms deviation being 0.052 Å. All the bond lengths and angles are normal and correspond to those observed in the related compounds (Wang *et al.*, 1988; Makhmudova *et al.*, 2000; Golenya *et al.*, 2007). In the crystal structure, intermolecular O1—H1···N2 and N1—H2···O2 hydrogen bonds (Table 1) link the molecules into a two-dimensional array in the *bc* plane (Fig. 2).

### Experimental

4-pyridinecarboxylic acid was dissolved in methanol (50 mL) and concentrated sulfuric acid (5 mL) was added drop wise into the reactor. The mixture was then stirred and refluxed for 3 h, after which time the solution was adjusted to pH 8 by the use of a 5% sodium carbonate aqueous solution. Methyl 4-pyridinecarboxylate was obtained by extraction with diethyl ether. Yield: 61.6%. A mixture of hydroxylamine hydrochloride and sodium hydroxide was added drop wise to the methanol solution of methyl 4-pyridinecarboxylate. The reaction was allowed to continue at room temperature for 72 h, when the mixture was acidified to pH 5.5 by 5% HCl solution. The solvent was removed *in vacuo* and the product was recrystallized from water to give pale-red crystals. Yield: 82%, M.pt. 421 K.

### Refinement

The H atoms were geometrically placed (O—H = 0.82 Å, N—H = 0.86 Å and C—H = 0.93 Å) and refined as riding with *U*_iso_(H) = 1.2-1.5*U*_eq_(parent atom).

### Crystal data

**Table:**

C_6_H_6_N_2_O_2_	*F*(000) = 288
*M**_r_* = 138.13	*D*_x_ = 1.468 Mg m^−^^3^
Monoclinic, *P*2_1_/*c*	Mo *K*α radiation, λ = 0.71073 Å
Hall symbol: -P 2ybc	Cell parameters from 1066 reflections
*a* = 4.8765 (5) Å	θ = 2.6–26.0°
*b* = 13.4476 (16) Å	µ = 0.11 mm^−^^1^
*c* = 9.6656 (11) Å	*T* = 298 K
β = 99.579 (1)°	Block, pale-red
*V* = 625.01 (12) Å^3^	0.35 × 0.24 × 0.15 mm
*Z* = 4	

### Data collection

**Table:**

Bruker SMART CCD area-detector diffractometer	1092 independent reflections
Radiation source: fine-focus sealed tube	769 reflections with *I* > 2σ(*I*)
graphite	*R*_int_ = 0.024
φ and ω scans	θ_max_ = 25.0°, θ_min_ = 2.6°
Absorption correction: multi-scan (*SADABS*; Sheldrick, 1996)	*h* = −5→5
*T*_min_ = 0.961, *T*_max_ = 0.983	*k* = −13→16
3030 measured reflections	*l* = −11→10

### Refinement

**Table:**

Refinement on *F*^2^	Primary atom site location: structure-invariant direct methods
Least-squares matrix: full	Secondary atom site location: difference Fourier map
*R*[*F*^2^ > 2σ(*F*^2^)] = 0.039	Hydrogen site location: inferred from neighbouring sites
*wR*(*F*^2^) = 0.115	H-atom parameters constrained
*S* = 1.05	*w* = 1/[σ^2^(*F*_o_^2^) + (0.0528*P*)^2^ + 0.208*P*] where *P* = (*F*_o_^2^ + 2*F*_c_^2^)/3
1092 reflections	(Δ/σ)_max_ < 0.001
92 parameters	Δρ_max_ = 0.15 e Å^−^^3^
0 restraints	Δρ_min_ = −0.16 e Å^−^^3^

### Fractional atomic coordinates and isotropic or equivalent isotropic displacement parameters (Å^2^)

**Table:**

	*x*	*y*	*z*	*U*_iso_*/*U*_eq_	
O2	0.1487 (3)	0.30086 (10)	0.06585 (14)	0.0430 (4)	
O1	−0.1208 (3)	0.35086 (10)	0.27794 (16)	0.0437 (5)	
H1	−0.0465	0.4055	0.2770	0.065*	
N1	0.0809 (4)	0.27699 (12)	0.28861 (17)	0.0364 (5)	
H2	0.1275	0.2453	0.3662	0.044*	
C1	0.2019 (4)	0.25531 (14)	0.1785 (2)	0.0324 (5)	
C2	0.4074 (4)	0.17092 (14)	0.1980 (2)	0.0331 (5)	
N2	0.7903 (4)	0.01429 (13)	0.2167 (2)	0.0477 (5)	
C6	0.5567 (5)	0.15118 (16)	0.0916 (2)	0.0420 (6)	
H6	0.5325	0.1905	0.0114	0.050*	
C3	0.4594 (6)	0.11050 (18)	0.3153 (2)	0.0570 (7)	
H3	0.3672	0.1214	0.3908	0.068*	
C5	0.7415 (5)	0.07300 (17)	0.1049 (2)	0.0462 (6)	
H5	0.8377	0.0606	0.0313	0.055*	
C4	0.6486 (6)	0.03405 (19)	0.3196 (3)	0.0659 (8)	
H4	0.6790	−0.0061	0.3990	0.079*	

### Atomic displacement parameters (Å^2^)

**Table:**

	*U*^11^	*U*^22^	*U*^33^	*U*^12^	*U*^13^	*U*^23^
O2	0.0581 (10)	0.0425 (9)	0.0303 (8)	0.0067 (8)	0.0127 (7)	0.0035 (6)
O1	0.0467 (9)	0.0354 (8)	0.0525 (10)	0.0019 (7)	0.0187 (7)	−0.0029 (7)
N1	0.0442 (11)	0.0354 (10)	0.0312 (9)	0.0040 (8)	0.0107 (8)	−0.0002 (7)
C1	0.0404 (12)	0.0305 (10)	0.0272 (10)	−0.0053 (9)	0.0084 (9)	−0.0039 (9)
C2	0.0382 (12)	0.0292 (10)	0.0325 (11)	−0.0044 (9)	0.0078 (9)	−0.0043 (8)
N2	0.0514 (12)	0.0356 (10)	0.0584 (12)	0.0025 (9)	0.0162 (10)	0.0023 (9)
C6	0.0448 (13)	0.0485 (14)	0.0341 (12)	0.0047 (11)	0.0103 (10)	0.0022 (9)
C3	0.0782 (19)	0.0518 (15)	0.0495 (14)	0.0206 (13)	0.0353 (13)	0.0163 (11)
C5	0.0455 (14)	0.0488 (14)	0.0469 (14)	0.0045 (11)	0.0156 (11)	−0.0046 (11)
C4	0.090 (2)	0.0537 (16)	0.0620 (17)	0.0249 (15)	0.0364 (16)	0.0262 (13)

### Geometric parameters (Å, °)

**Table:**

O2—C1	1.239 (2)	N2—C5	1.327 (3)
O1—N1	1.390 (2)	N2—C4	1.329 (3)
O1—H1	0.8200	C6—C5	1.377 (3)
N1—C1	1.332 (2)	C6—H6	0.9300
N1—H2	0.8600	C3—C4	1.378 (3)
C1—C2	1.505 (3)	C3—H3	0.9300
C2—C6	1.381 (3)	C5—H5	0.9300
C2—C3	1.384 (3)	C4—H4	0.9300
			
N1—O1—H1	109.5	C5—C6—H6	120.2
C1—N1—O1	119.96 (16)	C2—C6—H6	120.2
C1—N1—H2	120.0	C4—C3—C2	119.5 (2)
O1—N1—H2	120.0	C4—C3—H3	120.2
O2—C1—N1	122.57 (19)	C2—C3—H3	120.2
O2—C1—C2	121.35 (17)	N2—C5—C6	123.8 (2)
N1—C1—C2	116.07 (17)	N2—C5—H5	118.1
C6—C2—C3	116.7 (2)	C6—C5—H5	118.1
C6—C2—C1	118.33 (18)	N2—C4—C3	123.8 (2)
C3—C2—C1	124.92 (18)	N2—C4—H4	118.1
C5—N2—C4	116.4 (2)	C3—C4—H4	118.1
C5—C6—C2	119.7 (2)		
			
O1—N1—C1—O2	2.4 (3)	C1—C2—C6—C5	−178.48 (19)
O1—N1—C1—C2	−177.12 (15)	C6—C2—C3—C4	−1.3 (4)
O2—C1—C2—C6	6.1 (3)	C1—C2—C3—C4	178.6 (2)
N1—C1—C2—C6	−174.36 (18)	C4—N2—C5—C6	0.2 (3)
O2—C1—C2—C3	−173.7 (2)	C2—C6—C5—N2	−0.9 (3)
N1—C1—C2—C3	5.8 (3)	C5—N2—C4—C3	−0.1 (4)
C3—C2—C6—C5	1.4 (3)	C2—C3—C4—N2	0.6 (4)

### Hydrogen-bond geometry (Å, °)

**Table:**

*D*—H···*A*	*D*—H	H···*A*	*D*···*A*	*D*—H···*A*
O1—H1···N2^i^	0.82	1.92	2.721 (2)	166
N1—H2···O2^ii^	0.86	2.01	2.844 (2)	162

Symmetry codes: (i) −*x*+1, *y*+1/2, −*z*+1/2; (ii) *x*, −*y*+1/2, *z*+1/2.
